# The anti-phage defense system GAPS4 is a double-edged sword that sensitizes bacteria to DNA-damaging agents

**DOI:** 10.1093/nar/gkag1104

**Published:** 2026-11-10

**Authors:** Tridib Mahata, Katarzyna Kanarek, Anushree Haldar, Rameshkumar Marimuthu Ragavan, Guy Shur, Ruba Yehia, David Burstein, Yoni Haitin, Udi Qimron, Dor Salomon

**Affiliations:** Department of Clinical Microbiology and Immunology, Gray Faculty of Medical and Health Sciences, Tel-Aviv University, Tel-Aviv 6997801, Israel; Department of Clinical Microbiology and Immunology, Gray Faculty of Medical and Health Sciences, Tel-Aviv University, Tel-Aviv 6997801, Israel; Department of Clinical Microbiology and Immunology, Gray Faculty of Medical and Health Sciences, Tel-Aviv University, Tel-Aviv 6997801, Israel; Department of Clinical Microbiology and Immunology, Gray Faculty of Medical and Health Sciences, Tel-Aviv University, Tel-Aviv 6997801, Israel; The Shmunis School of Biomedicine and Cancer Research, George S. Wise Faculty of Life Sciences, Tel-Aviv University, Tel-Aviv 6997801, Israel; Department of Physiology and Pharmacology, Gray Faculty of Medical and Health Sciences, Tel-Aviv University, Tel-Aviv 6997801, Israel; The Shmunis School of Biomedicine and Cancer Research, George S. Wise Faculty of Life Sciences, Tel-Aviv University, Tel-Aviv 6997801, Israel; Department of Physiology and Pharmacology, Gray Faculty of Medical and Health Sciences, Tel-Aviv University, Tel-Aviv 6997801, Israel; Sagol School of Neuroscience, Tel-Aviv University, Tel-Aviv 6997801, Israel; Department of Clinical Microbiology and Immunology, Gray Faculty of Medical and Health Sciences, Tel-Aviv University, Tel-Aviv 6997801, Israel; Department of Clinical Microbiology and Immunology, Gray Faculty of Medical and Health Sciences, Tel-Aviv University, Tel-Aviv 6997801, Israel

## Abstract

The arms race between bacteria and bacteriophages (phages) gave rise to multiple layers of antagonistic mechanisms, many of which remain unexplored. Here, we investigated the widespread anti-phage defense system GAPS4. We found that GAPS4 forms a heterotetrameric complex containing active PD-(D/E)xK phosphodiesterase domains. It is triggered upon sensing DNA damage, leading to genomic DNA degradation and cell suicide. We further demonstrated that this activation mechanism renders GAPS4 a double-edged sword, sensitizing bacteria to various DNA-targeting antibacterial agents. Collectively, our findings reveal a possible evolutionary trade-off between anti-phage defense and immunity against interbacterial antagonism.

## Graphical Abstract

**Graphical Abstract:**
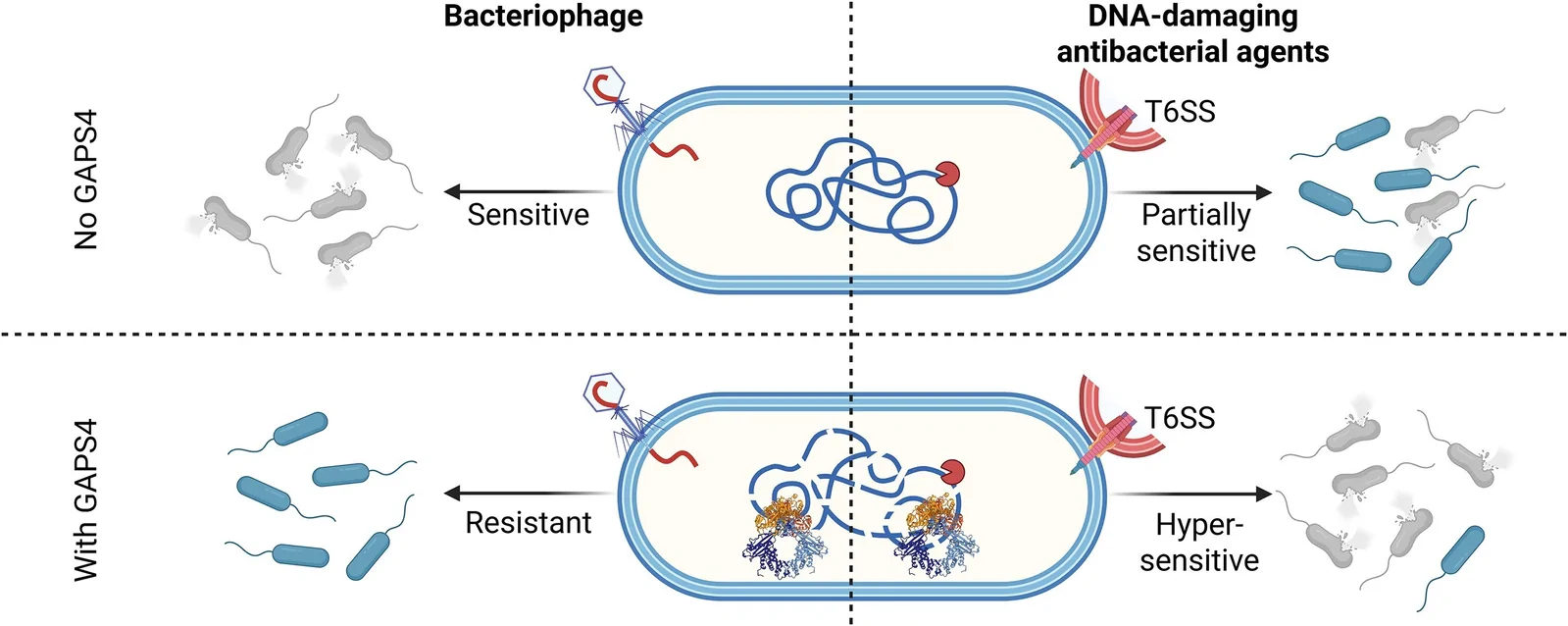

## Introduction

Bacteriophages (phages) prey on bacteria, leading to an evolutionary arms race in which bacteria evolve anti-phage defense mechanisms [1] and phages evolve countermeasures to antagonize them [2]. Anti-phage defense systems can recognize a conserved phage component, such as a phage protein or the incoming phage DNA, or a perturbation of a cellular process to mount either a restrictive or abortive response [3]. In the former, the bacterium specifically targets and restricts the incoming phage, whereas in the latter, it targets itself, often activating a suicide program to prevent phage proliferation [4].

In less than a decade, >200 types of anti-phage defense systems have been described [5, 6]. Many systems were identified based on their tendency to cluster in so-called “defense islands” [3, 7–10], which often reside within mobile genetic elements (MGEs) that spread defense arsenals across bacterial species [11–15].

We recently reported an anti-phage defense system named GAPS4, discovered in an MGE called the GMT island [13]. GMT islands, which are common in gamma-proteobacteria, employ a replicative transfer mechanism to mobilize diverse arsenals of anti-phage defense systems across bacterial genomes [13]. These arsenals enhance bacterial fitness during competitions against attacking phages. We showed that GAPS4 from *Vibrio parahaemolyticus* MAVP-R (GAPS4^MAVP-R^) protects against various coliphages when ectopically expressed in an *Escherichia coli* surrogate strain. Similar to most known anti-phage defense systems, the trigger, target, mechanism of action, and structure of GAPS4 remain unknown.

Here, we reveal that GAPS4 is a heterotetramer containing active PD-(D/E)xK phosphodiesterase domains. It senses DNA damage during phage infection and elicits degradation of bacterial DNA. This trigger mechanism renders GAPS4 a double-edged sword, hyper-sensitizing the bacteria to DNA-damaging antibacterial agents.

## Materials and methods

### Strains and media

For a complete list of strains used in this study, see Supplementary Table S1. *Escherichia coli* strains were grown in lysogeny broth (LB; 1% [w/v] tryptone, 0.5% [w/v] yeast extract, and 0.5% [w/v] NaCl) or 2xYT (1.6% [w/v] tryptone, 1% [w/v] yeast extract, and 0.5% [w/v] NaCl) at 37°C. *Vibrio parahaemolyticus* strains were grown in marine lysogeny broth (MLB; LB containing 3% [w/v] NaCl) and on marine minimal media (MMM) agar plates (1.5% [w/v] agar, 2% [w/v] NaCl, 0.4% [w/v] galactose, 5 mM MgSO_4_, 7 mM K_2_SO_4_, 77 mM K_2_HPO_4_, 35 mM KH_2_PO_4_, and 2 mM NH_4_Cl) at 30°C. Media were supplemented with 1.5% (w/v) agar to prepare solid plates. When required, media were supplemented with 35 or 10 µg/ml chloramphenicol (for *E. coli* and *V. parahaemolyticus*, respectively), 50 or 250 µg/ml kanamycin (for *E. coli* and *V. parahaemolyticus*, respectively), or 100 µg/ml ampicillin to maintain plasmids. To induce the expression from P*bad* promoters, 0.025%, 0.04%, or 0.2% (w/v) L-arabinose was added to the media, as indicated.

### Plasmid construction

For a complete list of plasmids and primers used in this study, see Supplementary Table S2 and Supplementary Table S3, respectively. Plasmids were constructed with standard molecular biology techniques using the Gibson Assembly method [16], unless otherwise mentioned. The Gibson Assembly master mix was obtained from NEB (E2611S). DNA fragments were amplified by PCR from bacterial genomic DNA or from bacteriophage genomic DNA, and Gibson Assembly ligations were carried out according to the manufacturer’s instructions.

### Plaque assays

The phages used in this study are listed in Supplementary Table S4. Phages were propagated on *E. coli* K12 MG1655 ΔRM [17]. To determine the effect of GAPS4^RiKo^ against coliphages T4, T5, T7, λ-vir, P1-vir, and 68 phages belonging to the BASEL collection [17], *E. coli* K12 MG1655 ΔRM strains harboring the pGAPS4^RiKo^ plasmid or an empty pC-0_v5 plasmid were grown overnight in LB supplemented with ampicillin at 37°C. Then, 200 μl of each bacterial culture were mixed with 7 ml of 0.7% (w/v) molten agar supplemented with 10 mM MgSO_4_ and 5 mM CaCl_2_. The mixtures were poured onto 1.5% (w/v) agar plates supplemented with ampicillin, 10 mM MgSO_4_, and 5 mM CaCl_2_, and the plates were left to dry. Tenfold serial dilutions of all the phages were prepared, and 7.5 μl of each dilution were spotted onto the dried plates. The plates were incubated overnight at 37°C. The following day, the plaques were counted, and the plaque-forming units (PFU) were calculated. For dilution spots in which no individual plaques were visible but a faint zone of lysis was observed, the dilution was considered as having ten plaques, as previously described [18]. A similar plaque assay was performed with *E. coli* MG1655 strains containing either pGAPS4^RiKo^ or pGAPS4^RiKo(D49A)^, and with *E. coli* BW25113 and its ∆*recA* derivative, using λ-vir. Plaque assays with λ-vir phage infecting *E. coli* MG1655 strains containing pBAD33.1-based plasmids encoding the indicated GAPS4^MAVP-R^ versions were performed on LB agar supplemented with chloramphenicol, 0.1% (w/v) L-arabinose (to induce the expression from the P*bad* promoter), and 10 mM MgSO_4_ at 37°C.

### Lysogenization and viability assays

*E. coli* MG1655 strains harboring pGAPS4^RiKo^ or pGAPS4^RiKo(D49A)^ were grown overnight in LB supplemented with ampicillin at 37°C. Overnight cultures were diluted 1:100 in 10 ml of fresh LB supplemented with ampicillin, 10 mM MgSO_4_, and 0.2% (w/v) D-maltose, and grown to an OD_600_ of ∼0.5. The cells were then infected with λ phage CI-857 containing a kanamycin-resistance cassette [19] at a multiplicity of infection (MOI) of ∼0.1 or ∼5, and incubated for 120 min at 30°C. Tenfold serial dilutions of these infected cultures were spotted onto 1.5% (w/v) agar plates supplemented with kanamycin and ampicillin (to select for lysogens) or only ampicillin (to determine the total number of viable cells), and the plates were incubated overnight at 30°C. The colonies were then counted, and the colony-forming units (CFU)/ml were calculated.

### Transformation into λ lysogen assays

*E. coli* BW25113 cells lysogenized with λ phage CI857 containing a kanamycin-resistance cassette were grown overnight in LB supplemented with kanamycin at 30°C. The cells were diluted 1:100 in 10 ml of fresh LB supplemented with kanamycin and grown at 30°C. Upon reaching an OD_600_ of ∼0.5, the cells were harvested, washed twice with chilled double-distilled H_2_O (DDW), and resuspended in 150 μl of chilled DDW. The cells were electroporated with equal molar amounts of pGAPS4^RiKo^ or pGAPS4^RiKo(D49A)^, and then diluted and spotted onto 1.5% (w/v) agar plate supplemented with kanamycin and ampicillin. The plates were incubated overnight at 30°C. The next day, the transformed colonies were counted, and the CFU/ml was calculated.

### Transduction assays

Transduction assays were carried out as previously described [20]. Briefly, *E. coli* BW25113 cells harboring the T7-packaging signal-containing pIYPE69 plasmid were grown overnight in LB supplemented with kanamycin at 37°C. The cells were diluted 1:100 in fresh LB supplemented with kanamycin and grown at 37°C. Upon reaching an OD_600_ of ∼0.5, the cells were infected with wild-type T7 phage at an MOI of 2. Infected cells were grown until lysis occurred (1.5–2 h). Then, 200 µl of chloroform were added, and the lysates were centrifuged to clear cell debris. The resulting supernatants contained a mixture of T7 phage particles with either a T7 genome or a pIYPE69 plasmid carrying a T7 packaging signal. For the transduction assay, *E. coli* BW25113 Δ*trxA* strains harboring pGAPS4^RiKo^ or pGAPS4^RiKo(D49A)^ were grown overnight in LB supplemented with ampicillin at 37°C. Overnight cultures were diluted 1:100 in 3 ml of fresh LB supplemented with ampicillin and grown to an OD_600_ of ∼0.5. The cells were then mixed at a 1:1 (v/v) ratio with tenfold serial dilutions of the prepared phage lysates described above. Transduction was carried out for 1 h at 37°C with shaking. Then, the cells were spotted onto 1.5% (w/v) agar plate supplemented with kanamycin and ampicillin, and incubated overnight at 37°C. The next day, the transduced colonies were counted, and the CFU/ml was calculated.

### *In vivo* DNA stability assays

To determine the effect of phage infection on DNA stability, *E. coli* MG1655 strains harboring pGAPS4^MAVP-R^, pGAPS4^MAVP-R(D59A)^, pGAPS4^RiKo^, or pGAPS4^RiKo(D49A)^ were grown overnight in LB supplemented with chloramphenicol (for GAPS4^MAVP-R^ plasmids) or ampicillin (for GAPS4^RiKo^ plasmids) and 0.2% (w/v) D-glucose at 37°C. Overnight cultures were washed twice, diluted 1:100 in 10 ml of fresh LB supplemented with the appropriate antibiotics, and grown for an hour at 37°C. L-arabinose (0.04% [w/v]) was added to the cultures to induce expression from the P*bad* promoter, and cells were grown for an additional hour at 37°C. The cells were then infected with λ-vir or Basel_01 phage at an MOI of ∼10, and the cultures were incubated for 90 min at 37°C with shaking. Then, cells equivalent to 1 OD_600_ units were collected from each sample, and genomic DNA was isolated using the Presto^™^ Mini gDNA isolation kit (Geneaid Biotech). Equal volumes of genomic DNA were then resolved on a 0.8% (w/v) agarose gel.

To determine the effect of nalidixic acid on bacterial genomic DNA in the presence of GAPS4^MAVP-R^, *E. coli* MG1655 strains harboring pGAPS4^MAVP-R^ or pGAPS4^MAVP-R(D59A)^ were grown overnight in LB supplemented with chloramphenicol and 0.2% (w/v) D-glucose at 37°C. Overnight cultures were washed twice, diluted 1:100 in 10 ml of fresh LB supplemented with chloramphenicol, and grown for 45 min at 37°C. L-arabinose (0.04% [w/v]) was added to the cultures to induce expression from the P*bad* promoter, and cells were grown for 45 additional minutes at 37°C. Next, nalidixic acid was added at a final concentration of 5 μg/ml, and the cultures were incubated for 90 min at 37°C with shaking. Genomic DNA was extracted from cells equivalent to 1.0 OD_600_ units and resolved on a 0.8% (w/v) agarose gel. A similar experiment was carried out to study the effect of nalidixic acid on bacterial genomic DNA in the presence of GAPS4^RiKo^, with *E. coli* MG1655 cells harboring pGAPS4^RiKo^ or pGAPS4^RiKo(D49A)^. Overnight cultures were diluted 1:100 in 10 ml of fresh LB supplemented with ampicillin and grown for 90 minutes at 37°C. Nalidixic acid was added at a final concentration of 5 μg/ml, and genomic DNA was extracted from cells equivalent to 1 OD_600_ units following incubation for 60, 120, and 180 min at 37°C with shaking, and resolved on a 0.8% (w/v) agarose gel.

### Microscopy

To visualize the effect of nalidixic acid on cellular DNA content in the presence of GAPS4^MAVP-R^ using fluorescence microscopy, *E. coli* MG1655 cells harboring pGAPS4^MAVP-R^ or pGAPS4^MAVP-R(D59A)^ were grown overnight at 37°C in LB supplemented with chloramphenicol and 0.2% (w/v) D-glucose. Overnight cultures were washed twice, then diluted 1:100 in 3 ml of fresh LB supplemented with chloramphenicol and incubated at 37°C for 45 min. Cells were then induced with 0.025% L-arabinose and incubated for 45 additional minutes. Next, nalidixic acid was added at a final concentration of 5 μg/ml, and the cells were incubated for 75 min. Following incubation, the cells were washed twice with M9 media and stained for 10 min at room temperature with Hoechst 33342 (Invitrogen) DNA dye at a final concentration of 1 μg/ml. The cells were then washed twice and resuspended in 10 μl of M9 media. To visualize the cell wall of *E. coli*, cells were incubated with Wheat Germ Agglutinin Alexa Fluor 488 Conjugate (Biotium; Catalog no. 29022-1) at a final concentration of 0.1 mg/ml and incubated for 10 min at room temperature. Next, 2 μl of each cell suspension were spotted onto 1% (w/v) LB-agarose pads, which were placed face-down in 35 mm glass-bottom Cellview cell culture dishes. The imaging was carried out using a Nikon Eclipse Ti2-E inverted motorized microscope equipped with a CFI PLAN apochromat DM 100X oil lambda PH-3 (NA, 1.45) objective lens, Lumencor SOLA SE II 395 light source, and DS-QI2 mono cooled digital microscope camera (16 MP). An ET-EGFP filter set (#49002) was used to visualize the Alexa Fluor 488 signal, and an ET-DAPI (#49028) was used to visualize the Hoechst signal. The captured images were further processed using the Fiji ImageJ suite [21].

### Bacterial competition assays

Bacterial competition assays were performed as previously described [22], with minor modifications. Briefly, bacteria were grown overnight in appropriate media supplemented with antibiotics to maintain plasmids: *V. parahaemolyticus* attacker strains in MLB and *E. coli* BW25113^tetR^ prey in 2xYT. Overnight cultures were normalized to OD_600_ = 0.5 and mixed at a 1:1 ratio (attacker:prey). Triplicates of the mixtures were spotted onto MLB agar plates supplemented with 0.1% (w/v) L-arabinose and incubated for 4 h at 30°C. Samples from the 0-h timepoint and samples recovered from the assay plates after 4 h were plated on selective media to quantify the viability (CFU) of the prey strains. The experiments were performed three times with similar results; results from a representative experiment are shown.

### Antibiotic sensitivity assays

Disk diffusion assays were performed using the soft agar overlay technique. *E. coli* BW25113^tetR^ strains harboring the indicated plasmids were grown overnight in 2xYT media supplemented with antibiotics to maintain the plasmids. Overnight cultures were then diluted 1:100 into fresh media and grown to OD_600_ ∼0.9. Then, the cultures were mixed to a final OD_600_ of 0.08 or 0.05 (for 130 mm square plates or 90 mm round plates, respectively) with 0.5% (w/v) molten agar. The mixtures were poured onto 1.5% (w/v) LB agar plates as described above, and the plates were left to dry for an hour at room temperature. Next, sterile Whatman paper discs (5 mm diameter) were placed on the surface. Each disc was spotted with 7 µl of antibiotic solution, delivering final amounts of 30 µg nalidixic acid, 10 µg gentamicin, 5 µg trimethoprim, 50 µg rifampicin, 300 µg novobiocin, 10 µg ciprofloxacin, or 2 µg mitomycin C. The plates were incubated at 37°C, and the diameter of each zone of inhibition around a disc was measured after 16 h using the ImageJ software [21]. Each assay included three biological replicates. The experiments were performed at least twice with similar results; results from a representative experiment are shown.

For spotting assays, *E. coli* MG1655 cells containing pGAPS4^MAVP-R^ or pGAPS4^MAVP-R(D59A)^ were grown overnight in LB supplemented with chloramphenicol and 0.2% (w/v) D-glucose at 37°C. Overnight cultures were washed twice, diluted 1:100 in 3 ml of fresh LB supplemented with chloramphenicol, and grown for 45 min at 37°C. L-arabinose (0.04% [w/v]) was added to the cultures to induce expression from the P*bad* promoter, and the cells were grown for 45 additional minutes at 37°C. Then, tenfold serial dilutions were spotted onto 1.5% (w/v) agar plate supplemented with chloramphenicol, L-arabinose (0.04% [w/v]), and the indicated antibiotic concentrations. The plates were incubated overnight at 37°C.

### SOS response activation assays

To evaluate the activation of the SOS response during exposure to antibiotics, *E. coli* BW25113^tetR^ strains harboring a plasmid constitutively expressing the bacterial Lux operon [23] and a plasmid containing a GFP reporter under the control of the *E. coli recA* SOS-responsive promoter [24] were used as described above in a disk diffusion assay. Fluorescent signals were acquired using Fusion FX6 (Vilber Lourmat).

### Protein purification

For purification of the GAPS4^MAVP-R^ complex, pMBP- GAPS4^MAVP-R^ was transformed into *E. coli* BL21 (DE3) cells and grown in 2 l of 2xYT supplemented with 50 μg/ml kanamycin at 37°C until they reached an OD_600_ ∼0.5. Then, GAPS4 expression was induced by adding 0.5 mM isopropyl β-D-1-thiogalactopyranoside (IPTG), and the cells were grown at 16°C for 18 h. The cells were then harvested and resuspended in lysis buffer (20 mM HEPES–NaOH pH 7.5, 300 mM NaCl, 10 mM imidazole, 1 mM dithiothreitol [DTT], 1 mM phenylmethylsulfonyl fluoride [PMSF], 10% [v/v] glycerol). Sonication was used to lyse the cells, and the supernatant was collected after centrifugation. The cleared supernatant was loaded onto a 1 ml His-Trap column (GE Healthcare) and washed with 25 column volumes of wash buffer (20 mM HEPES–NaOH pH 7.5, 300 mM NaCl, 10 mM imidazole, 1 mM DTT, 1 mM PMSF, 5% [v/v] glycerol). The bound proteins were then eluted with elution buffer (20 mM HEPES–NaOH pH 7.5, 300 mM NaCl, 300 mM imidazole, 1 mM DTT, 5% [v/v] glycerol) and dialyzed overnight at 4°C against dialysis buffer (20 mM HEPES–NaOH pH 7.5, 300 mM NaCl, 10 mM imidazole, 1 mM DTT, 5% [v/v] glycerol) in the presence of TEV protease to remove the His-Maltose binding protein (MBP) tag. The cleaved proteins were again passed through a 1 ml His-Trap column, and the flow-through containing the cleaved proteins was collected. The proteins were concentrated using Amicon Ultra-4 Centrifugal Filter Units (10 kDa columns) and further purified in a Superdex 200 Increase 10/300 size-exclusion chromatography column (GE Healthcare) equilibrated with protein buffer (20 mM HEPES–NaOH pH 7.5, 150 mM NaCl, 1 mM DTT). The fractions containing the protein were pooled, concentrated using Amicon Ultra-4 Centrifugal Filter Units (10-kDa columns), snap-frozen, and stored at −80°C.

### SEC-MALS analyses

Experiments were carried out using an analytical size-exclusion chromatography (SEC) column (Superdex 200 10/300 GL), pre-equilibrated with SEC buffer (20 mM HEPES–NaOH pH 7.5, 150 mM NaCl, 1 mM DTT). Purified GAPS4^MAVP-R^ complex (1–2 mg/ml; 50 µl) was injected onto an HPLC system connected in-line to an 8-angle multi-angle light-scattering (MALS) detector and a differential refractive index (RI) detector (Wyatt Technology, CA, USA). RI and MALS data were analyzed using ASTRA software (Wyatt Technology) to determine the absolute molecular mass of the complex.

### Gel shift assays

DNA binding was determined *in vitro* using a 2054-bp PCR product amplified from *E. coli* MG1655 genomic DNA with Cheetah Taq DNA polymerase (Biotium) and purified with the Gel/PCR DNA Fragments Extraction Kit (Geneaid Biotech).

DNA binding was assessed in 10 µl reactions containing 200 ng of the purified PCR amplicon and the indicated amount of the purified GAPS4^MAVP-R^ complex described above or bovine serum albumin (BSA, Sigma, A9647). The final reaction buffer included 20 mM HEPES-NaOH (pH 7.5), 25 mM NaCl, 10 mM EDTA, and 1 mM DTT. The reaction mixtures were incubated at 37°C for 60 min. Reactions were terminated by the addition of 1 µl of 0.5 M EDTA (pH 8.0) and mixed with 6x ExcelDye™ DNA Loading Dye (DL3000) before being resolved on 0.8% agarose gels. Gels were imaged using a Fusion FX6 imaging system (Vilber Lourmat).

### AlphaFold structure predictions

GAPS4 complex structures were predicted using AlphaFold3 [25] (https://alphafoldserver.com/). The best model was selected and visualized using ChimeraX [26] version 1.7.1 together with its Predicted Aligned Error (PAE) plot.

### Identification of GAPS4 homologs

Each of the GAPS4a^MAVP-R^ and GAPS4b^MAVP-R^ sequences (WP_055466293.1 and WP_055466294.1, respectively) was used to build a profile Hidden Markov Model (pHMM) using hmmbuild from the HMMER suite [27] (version 3.4, Aug 2023). These pHMMs were used to search all bacterial genomes from the NCBI GenBank Whole Genome Sequencing (WGS) database [28] (downloaded on March 14, 2020) to detect adjacent and fused GAPS4a and GAPS4b proteins. The concatenated and fused sequences were clustered using CD-HIT [29] with 0.9 global sequence identity. To further de-duplicate sequences using taxonomy, up to one sequence per order per cluster was selected, and the rest were discarded. The remaining sequences were aligned using MAFFT [30] (mafft-linsi), which was then used to construct a maximum-likelihood phylogenetic tree using RAxML [31] (version 8.2.12) with the PROTGAMMALG evolutionary model. The tree was visualized using the interactive Tree Of Life (iTOL) [32] web server.

### Illustration of conserved residues using WebLogo

The protein sequences of GAPS4a and GAPS4b homologs were aligned using CLUSTAL Omega [33] in MEGA11 [34]. Aligned columns not found in representative proteins (WP_055466293.1 and WP_055466294.1) were discarded. The conserved residues were illustrated using the WebLogo server (weblogo.berkeley.edu) [35].

## Results

### GAPS4 represents a widespread family of anti-phage defense systems

GAPS4^MAVP-R^ comprises two genes encoding GAPS4a and GAPS4b (Fig. 1a; WP_055466293.1 and WP_055466294.1, respectively). To determine whether both genes are required for anti-phage protection, we examined the ability of each gene to confer resistance to lytic phage λ attacks. We found that neither GAPS4a nor GAPS4b protects *E. coli* in the absence of its counterpart, indicating that both are essential components of the defense system (Fig. 1b).

**Figure 1.**
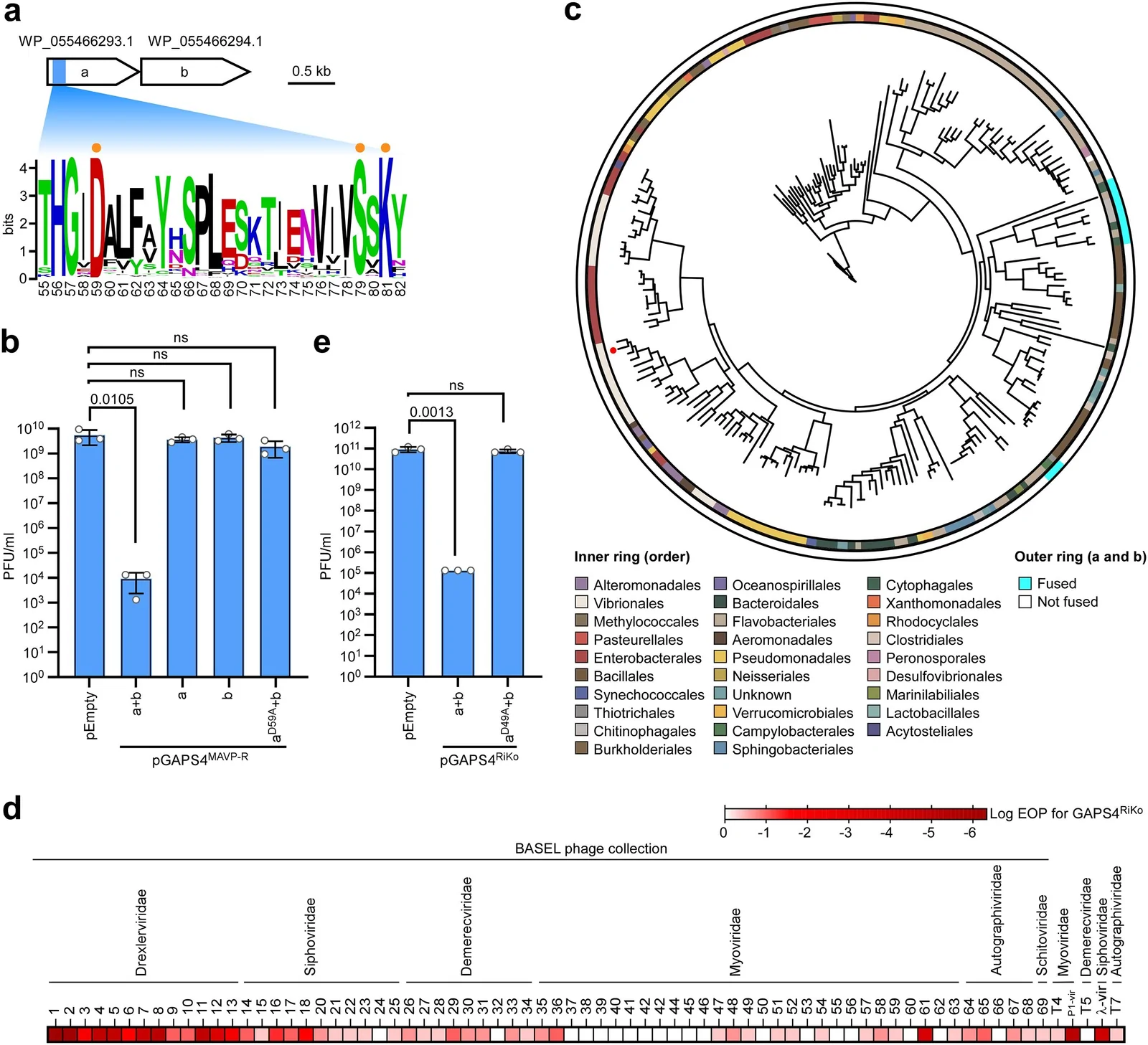
GAPS4 is a widespread family of anti-phage defense systems with a PD-(D/E)xK phosphodiesterase active domain. (**a**) Schematic representation of the GAPS4 system. The conservation logo of the predicted PD-(D/E)xK phosphodiesterase domain active site found in GAPS4 homologs (denoted by orange circles) is shown below. The residue numbers correspond to positions in GAPS4a^MAVP-R^ (WP_055466293.1). (**b** and **e**) Plaque-forming units (PFUs) of λ-vir phage upon infection of *E. coli* strains containing an empty plasmid (pEmpty) or a plasmid for the arabinose-inducible expression of the indicated GAPS4^MAVP-R^ version (b) or the expression of the indicated GAPS4^RiKo^ from its native promoter (e). Statistical significance between samples was calculated using one-way ANOVA with Dunnett multiple comparisons correction; ns, no significant difference (*P* > 0.05). Data are shown as mean ± SD of three independent experiments. (**c**) Phylogenetic distribution of GAPS4 homologs. The bacterial order is denoted by color. The outer ring denotes GAPS4 homologs that are naturally fused into a single gene. The evolutionary history was inferred using RAxML on a concatenated alignment of GAPS4a and GAPS4b along with fused GAPS4a-b proteins. GAPS4^MAVP-R^ is denoted with a red circle. (**d**) The efficiency of plating (EOP; expressed as the log difference in plaque numbers) was determined for *E. coli* harboring a low-copy plasmid encoding GAPS4^RiKo^ with its native promoter, when challenged with 73 coliphages, compared with *E. coli* containing an empty plasmid. The data shown are the average of three independent experiments.

Computational analyses revealed 1651 non-redundant GAPS4 homologs (comprising GAPS4a and GAPS4b in tandem or fused as a single gene) across different bacterial orders, including Gram-negative and Gram-positive bacteria inhabiting diverse ecological niches (Fig. 1c, Supplementary Dataset S1, and File S1). To determine whether GAPS4 homologs also function as anti-phage defense systems, we subcloned a GAPS4 homolog from *E. coli* strain RiKo 2299/09 (GAPS4^RiKo^; WP_028132563.1 and WP_028132564.1; 30.63% identity with 88% query coverage and 25% identity with 97% query coverage compared to GAPS4a^MAVP-R^ and GAPS4b^MAVP-R^, respectively), together with its native promoter, into a low-copy plasmid. We reasoned that a homolog from *E. coli* would be a biologically relevant system to investigate the protective effect of GAPS4 against coliphages. Indeed, we observed that a phage-sensitive, surrogate *E. coli* strain harboring the GAPS4^RiKo^-encoding plasmid became resistant to attacks by multiple phages from diverse families compared to *E. coli* containing an empty plasmid, as evidenced by a reduction of up to six orders of magnitude in the number of visible plaques that developed on a lawn of bacteria (i.e., the efficiency of plating; EOP) (Fig. 1d).

Amino acid conservation analyses of GAPS4a and GAPS4b homologs (Supplementary Fig. S1 and Supplementary Fig. S2) implied that GAPS4a belongs to a divergent family of PD-(D/E)xK phosphodiesterases with the non-canonical conserved motif HGxD-SxK [36, 37] at its N-terminus (Fig. 1a). PD-(D/E)xK phosphodiesterases comprise a large superfamily of nucleases and phosphodiesterases with a defined core fold and sequence motif, but minimal sequence conservation outside this motif [38, 39]. In agreement with this prediction, substitution of the conserved aspartic acid in position 59 with alanine abrogated GAPS4^MAVP-R^-mediated protection against lytic phage λ attacks (Fig. 1b). The respective conserved aspartic acid in GAPS4^RiKo^ (position 49) was also required for GAPS4^RiKo^-mediated protection (Fig. 1e). Collectively, our results indicate that GAPS4 is a widespread family of anti-phage defense systems with a predicted PD-(D/E)xK phosphodiesterase active domain.

### GAPS4 is a heterotetrameric complex

The results above indicate that both GAPS4 components are essential for its activity. Therefore, we hypothesized that GAPS4a and GAPS4b form a complex. Indeed, when we purified GAPS4a^MAVP-R^ using size-exclusion chromatography (SEC) from an *E. coli* strain expressing the GAPS4^MAVP-R^ operon (GAPS4a and GAPS4b), GAPS4b^MAVP-R^ co-purified with it, supporting our hypothesis (Fig. 2a). Notably, the SEC elution profile suggested a possible higher oligomeric state of the GAPS4 complex. To evaluate the stoichiometry and absolute molar mass of the purified GAPS4 complex, we used size-exclusion chromatography multi-angle light scattering (SEC-MALS). As shown in Fig. 2b, we observed a mono-dispersed complex with a calculated mass of 164.3 ± 11.5 kDa, corresponding with the predicted mass of a heterotetramer comprising two GAPS4a molecules and two GAPS4b molecules (160.8 kDa).

**Figure 2.**
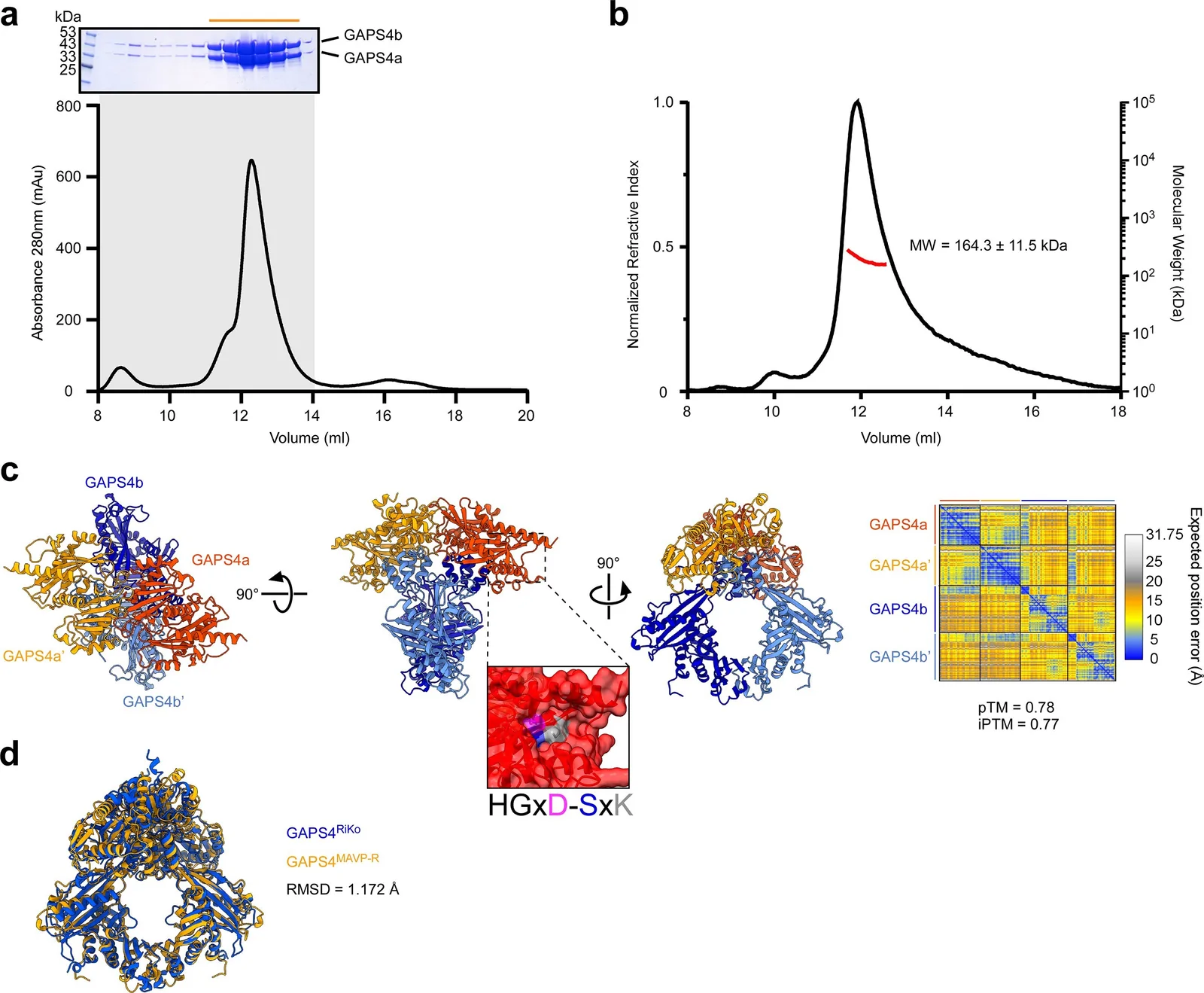
GAPS4 is a heterotetramer complex. (**a**) Size-exclusion chromatography (SEC) purification of GAPS4a^MAVP-R^. Coomassie blue-stained SDS–PAGE of the eluted fractions denoted in gray is shown above. The fractions indicated by the orange line were pooled and used for subsequent analyses. (**b**) SEC-MALS analysis of purified GAPS4^MAVP-R^. The black line represents the normalized refractive index, and the red line represents the calculated molecular weight (MW) in kDa. (**c**) Structural model of GAPS4^MAVP-R^ heterotetramer predicted by AlphaFold3. The AlphaFold Predicted Aligned Error (PAE) plot is shown on the right. The color key indicates the expected position error for each residue pair in Å units. Blue represents low predicted errors, indicating high confidence in the relative positions of those residues. The orange color indicates higher predicted errors, suggesting lower confidence. The rectangular inset is a close-up view of the predicted PD-(D/E)xK active site in GAPS4a. (**d**) Superimposition of AlphaFold3-predicted GAPS4^RiKo^ and GAPS4^MAVP-R^ complexes.

Consistent with the SEC-MALS results, the AlphaFold3 analysis predicts, with high confidence, that GAPS4a and GAPS4b of GAPS4^MAVP-R^ form a heterotetramer with a 2:2 stoichiometry (Fig. 2c). In this model, the two GAPS4b subunits form a ring-like structure, and the GAPS4a subunits are docked onto α-helices-comprising globular domains in the GAPS4b subunits. The predicted active sites of the GAPS4a subunits, comprising the conserved HGxD-SxK motif, face outwards to opposite sides of the GAPS4b ring (Fig. 2c and Supplementary File S2). A similar complex is predicted for GAPS4^RiKo^ (Fig. 2d and Supplementary File S3).

### GAPS4 induces DNA degradation and cell death upon phage infection

Our analyses indicated that GAPS4 protects *E. coli* against several lytic phages, including λ-vir, an obligate lytic variant of the λ phage (Fig. 1) [13]. To determine whether GAPS4 can also protect bacteria against a lysogenic phage, we challenged *E. coli* cells harboring either a plasmid-borne GAPS4^RiKo^ or its inactive mutant GAPS4^RiKo(D49A)^ with λ CI-857 phage carrying a kanamycin-resistance cassette, under lysogenization-favoring conditions (CI-857 carries a temperature-sensitive cI variant, thus forming stable lysogens at temperatures below 37°C [40]). We obtained ∼100- to 1000-fold fewer lysogens in the presence of wild-type GAPS4^RiKo^, regardless of whether bacteria were challenged with the phage at a MOI of 0.1 or 5 (Fig. 3a). These results confirm that GAPS4 provides protection against both lytic and lysogenic phage attacks.

**Figure 3.**
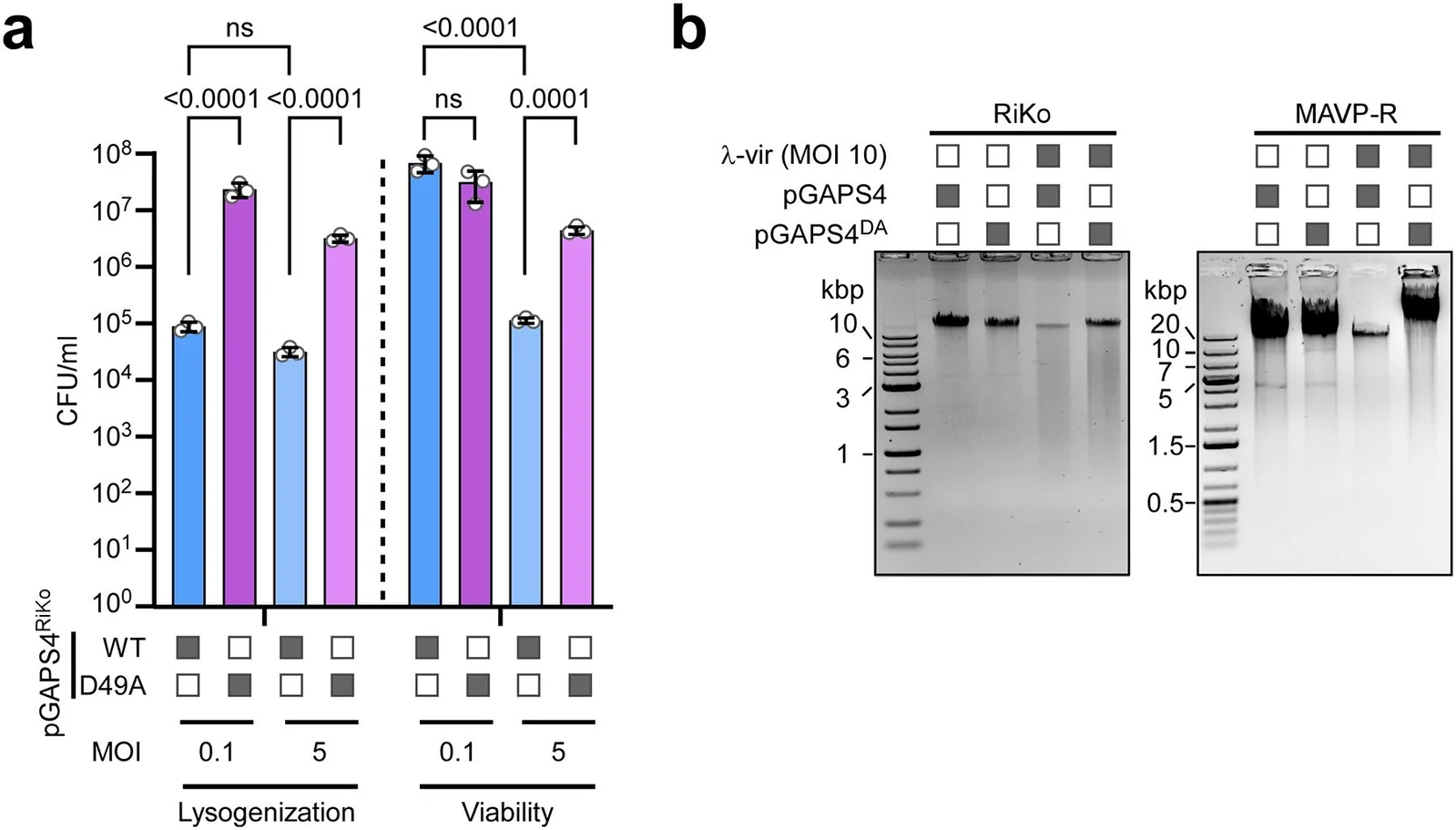
GAPS4 degrades DNA upon λ-vir phage infection. (**a**) Lysogenization and viability of *E. coli* strains containing the indicated GAPS4^RiKo^ upon challenge with λ CI-857 phage containing a kanamycin-resistance cassette under lysogenization-favorable conditions (30°C). After infection, bacteria were plated on agar plates containing kanamycin to select for lysogens and on plates without kanamycin to assess viability. Statistical significance between samples was assessed using a two-way ANOVA with Tukey’s multiple-comparison correction on log-transformed data. Data are shown as mean ± SD of three biological samples. (**b**) Genomic DNA stability after the addition of λ-vir phage to the media of *E. coli* cultures expressing the indicated GAPS4 variant from an arabinose-inducible plasmid (MAVP-R) or from its natural promoter (RiKo); MOI, multiplicity of infection. The experiments were repeated three times with similar results. Results from a representative experiment are shown.

GAPS4’s ability to protect against a lysogenic phage prompted us to investigate whether it restricts the incoming phage or induces bacterial cell suicide upon invasion. Although we did not detect a significant change in bacterial viability upon phage challenge at MOI 0.1 (when < 10% of bacteria are exposed to phage attack), we noted a significant reduction in viability (∼40-fold) at MOI 5 (when most bacteria are exposed to phage attack) in the presence of GAPS4^RiKo^ (Fig. 3a). If the system merely restricted the incoming phage rather than induced cell suicide, we would expect to observe minimal loss of viability. Therefore, these results suggest that GAPS4 induces cell suicide upon lysogenizing phage attack.

Many members of the PD-(D/E)xK phosphodiesterase superfamily target DNA [38]. Therefore, we hypothesized that GAPS4 induces cell suicide by targeting the bacterial genomic DNA upon phage infection. In support of this notion, we observed genomic DNA degradation upon infection of *E. coli* harboring GAPS4^RiKo^ or GAPS4^MAVP-R^ with the lytic phage λ-vir (Fig. 3b). Genomic DNA degradation was also apparent upon infection with the lytic phage BASEL_01 in the presence of GAPS4^MAVP-R^ (Supplementary Fig. S3). In all cases, genomic DNA degradation was dependent on the intact PD-(D/E)xK active site.

### GAPS4 is a sentinel of DNA integrity

Following these results, we sought to identify the trigger that induces GAPS4-mediated DNA degradation. Despite repeated attempts, we were unable to isolate escape phages that bypass GAPS4-mediated defense, leading us to hypothesize that GAPS4 senses not a phage protein but the injection of phage DNA or a host-cell perturbation that occurs during infection. Because we did not detect GAPS4-mediated cell death upon the presence of lysogenized λ phage DNA in the bacterial genome (Supplementary Fig. S4a) or upon plasmid DNA injection by a transducing phage T7 particle (Supplementary Fig. S4b), we reasoned that GAPS4 is not triggered by the phage-mediated injection process or its DNA sequence but rather by a host cell perturbation.

To mimic potential phage-induced perturbations of host cellular processes, we employed various compounds that inhibit DNA synthesis (trimethoprim), replication (novobiocin, nalidixic acid, and ciprofloxacin), transcription (rifampicin), or translation (gentamicin). When we determined the susceptibility of *E. coli* strains harboring wild-type or mutant GAPS4^RiKo^ to the antibacterial compounds, the wild-type GAPS4^RiKo^ specifically sensitized bacteria to nalidixic acid and ciprofloxacin, quinolone class gyrase inhibitors predicted to induce DNA breaks [41], as evidenced by a larger inhibition zone in a disc diffusion assay (Fig. 4a). Sensitivity to nalidixic acid was also observed with GAPS4^MAVP-R^ (Fig. 4b). Importantly, exposure to nalidixic acid in the presence of GAPS4^RiKo^ or GAPS4^MAVP-R^ led to degradation of the bacterial genomic DNA, similar to the effect of phage infection (Fig. 4c–e).

**Figure 4.**
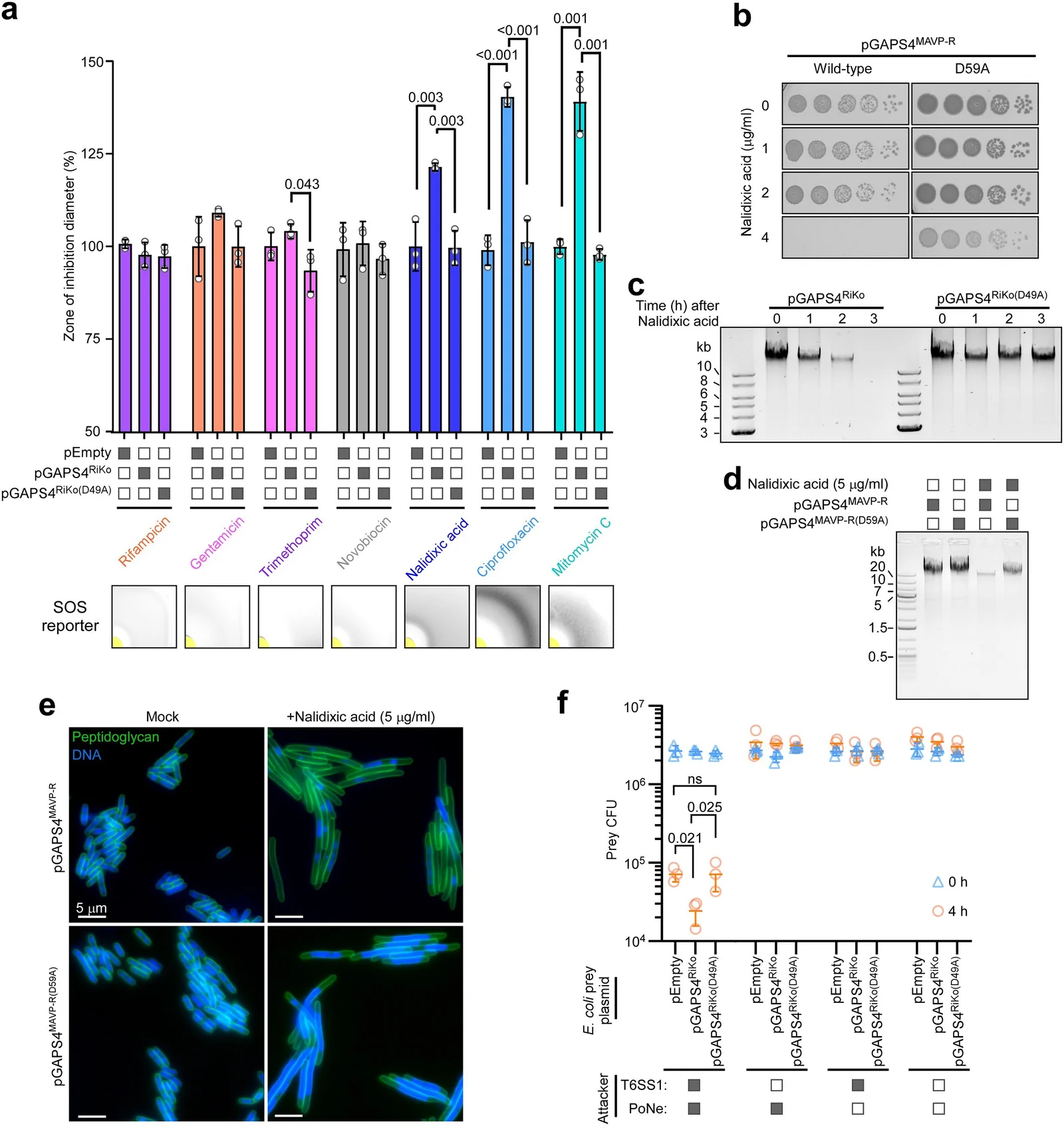
GAPS4 is activated by DNA damage. (**a**) Sensitivity of *E. coli* strains harboring an empty plasmid (pEmpty) or a plasmid expressing GAPS4^RiKo^ (pGAPS4^RiKo^) or its mutant (pGAPS4^RiKo(D49A)^) to the indicated compounds, measured as the zone of inhibition diameter around a filter disc containing the compound. Statistical significance between samples was assessed using one-way ANOVA with Tukey’s multiple-comparison correction. Data are shown as mean ± SD of three biological replicates. Fluorescence signals from *E. coli* strains harboring a plasmid encoding an SOS GFP reporter, measured in a similar disk diffusion assay, are shown below. Zone of inhibition quadrants from representative images are shown; the disks are indicated in yellow. (**b**) Growth of *E. coli* strains expressing the wild-type GAPS4^MAVP-R^ or its inactive mutant (D59A) from an arabinose-inducible plasmid spotted in 10-fold dilutions on agar plates supplemented with 0.04% (w/v) L-arabinose and the indicated concentration of nalidixic acid. (**c**) Genomic DNA stability after the addition of nalidixic acid (5 µg/ml) to the media of *E. coli* cultures containing the indicated GAPS4^RiKo^ on a plasmid. (**d**) Genomic DNA stability after the addition of nalidixic acid (5 µg/ml) to the media of *E. coli* cultures expressing the indicated GAPS4^MAVP-R^ variant from an arabinose-inducible plasmid. (**e**) Representative fluorescence microscopy images of *E. coli* strains expressing the indicated GAPS4^MAVP-R^ variant from an arabinose-inducible plasmid after 75 min of incubation with 5 µg/ml nalidixic acid. Peptidoglycan (green) and DNA (blue) are visualized; scale bar: 5 μm. (**f**) Viability counts (colony forming units; CFU) of *E. coli* prey strains containing the indicated plasmids before (0 h) and after (4 h) co-incubation with *V. parahaemolyticus* attacker strains lacking their endogenous T6SS1 antibacterial effectors, with either a functional (full square) or inactive (empty square) T6SS1, containing either an empty plasmid or a plasmid for expression of the DNase T6SS1 effector PoNe together with its cognate immunity gene and VgrG1b loading platform. Statistical significance between samples at the 4 h time point was assessed using one-way ANOVA with Tukey’s multiple-comparison correction on log-transformed data. Data are shown as mean ± SD of three biological replicates. In a and f: ns, no significant difference (*P* > 0.05). Additional ns comparisons were omitted for clarity. The experiments were repeated three times with similar results. Results from a representative experiment are shown.

Because novobiocin, a gyrase inhibitor acting through a mechanism distinct from that of nalidixic acid and ciprofloxacin, did not elicit a similar GAPS4-mediated effect, we hypothesized that DNA damage, rather than gyrase inhibition, accounted for the observed GAPS4-mediated sensitivity. To test this, we also examined the susceptibility of the *E. coli* strains to mitomycin C (MMC), a DNA-damaging agent that forms interstrand cross-links. GAPS4 sensitized the bacteria to MMC (Fig. 4a). Importantly, only the three compounds that elicited GAPS4-mediated sensitivity also triggered the SOS response, a gene expression program activated in cells undergoing DNA damage [42], as determined by monitoring the induction of a fluorescent SOS reporter in bacteria exposed to the examined compounds in a disk diffusion assay similar to the one used to determine antibiotic sensitivity (Fig. 4a).

It was previously suggested that the SOS response upregulates anti-phage defenses [43]. Therefore, we set out to examine whether GAPS4 is activated by the SOS response rather than by DNA damage per se. To this end, we monitored the ability of GAPS4^RiKo^, controlled by its natural promoter, to mount an anti-phage defense against a λ-vir attack in the absence of the DNA damage sensor *recA*. Since RecA is required for SOS response activation [42], GAPS4-mediated protection in its absence would suggest that GAPS4 is not activated by the SOS response. Indeed, we did not observe an effect of *recA* deletion on the anti-phage defense mediated by GAPS4 (Supplementary Fig. S5), further supporting the notion that GAPS4 is triggered by DNA damage. Nevertheless, we cannot rule out the possibility that *recA*-independent damage responses indirectly activate GAPS4.

### GAPS4 sensitizes bacteria to antibacterial agents that induce DNA damage

Various mechanisms of antibacterial antagonism can induce DNA damage. For example, type VI secretion systems (T6SSs) are offensive antibacterial weapons that deliver toxic proteins, called effectors, directly into neighboring target cells [44]. Because many T6SS effectors are DNases [45, 46], we hypothesized that if GAPS4 is triggered by DNA damage, then it can hyper-sensitize recipient bacteria to T6SS-mediated DNase attacks. To test this, we monitored the viability of *E. coli* prey cells harboring wild-type or mutant forms of GAPS4^RiKo^ after 4 h of co-incubation with attacking bacteria that specifically deliver a single T6SS DNase effector, PoNe (WP_029857615.1) [47]. In agreement with our hypothesis, prey cells harboring the wild-type GAPS4^RiKo^ were more susceptible to PoNe-mediated toxicity, as evidenced by ∼3-fold decrease in viability compared to prey cells harboring GAPS4^RiKo(D49A)^ or an empty plasmid (Fig. 4f). These results further support the conclusion that GAPS4 is triggered by DNA damage.

To further examine whether GAPS4 sensitizes bacteria to T6SS-mediated attacks, we employed the same experimental setup as above, using attacking bacteria that deliver other T6SS effectors, RIX^Vcamp^ (WP_005534959.1) or Rte1 (WP_006958655.1). Although their mechanisms of action and targets within the prey cell remain unknown, both effectors are predicted to act in the prey cytoplasm [48]. As shown in Supplementary Fig. S6, prey cells harboring the wild-type GAPS4^RiKo^ were more susceptible to RIX^Vcamp^-mediated toxicity, as evidenced by ∼4-fold decrease in viability compared to prey cells harboring GAPS4^RiKo(D49A)^ or an empty plasmid. However, prey cells harboring the wild-type GAPS4^RiKo^ were not more susceptible to Rte1-mediated toxicity. Therefore, GAPS4 sensitizes bacteria to specific T6SS effectors, but not to T6SS-mediated intoxication in general. Taken together, these results suggest that GAPS4 is a double-edged sword that protects its host from invading phages while sensitizing it to certain forms of antibacterial antagonism.

### GAPS4 binds DNA

Because our results suggest that GAPS4 senses DNA damage and elicits DNA degradation, we reasoned that it should be able to bind DNA. To test this, we performed a gel shift assay to monitor the interaction of the purified GAPS4^MAVP-R^ complex (Fig. 2) with a double-stranded PCR product. As shown in Fig. 5a, the addition of increasing amounts of GAPS4, but not of a bovine serum albumin (BSA) used as a control, resulted in reduced mobility of the PCR product, indicative of DNA binding; notably, when 5 µg of the GAPS4 complex were added, the DNA did not migrate out of the loading well (Fig. 5a).

**Figure 5.**
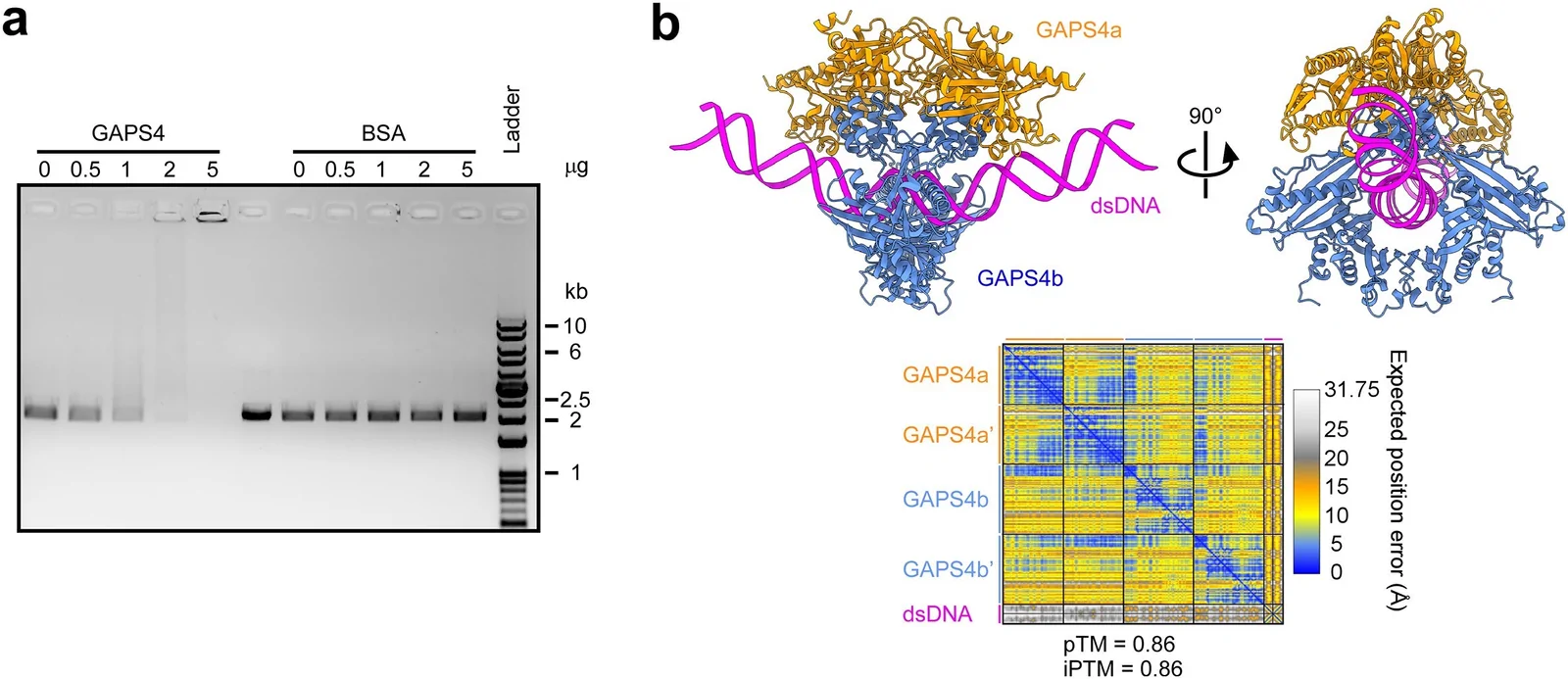
GAPS4 binds DNA. (**a**) Agarose gel shift assay using the purified GAPS4^MAVP-R^ complex or BSA and a PCR amplification product. The experiment was repeated three times with similar results. Results from a representative experiment are shown. (**b**) Structural model of GAPS4^MAVP-R^ heterotetramer with a random dsDNA molecule predicted by AlphaFold3 (top). The AlphaFold Predicted Aligned Error (PAE) plot is shown below. The color key indicates the expected position error for each residue pair in Å units. Blue represents low predicted errors, indicating high confidence in the relative positions of those residues. The orange color indicates higher predicted errors, suggesting lower confidence.

We were unable to purify stable GAPS4a and GAPS4b individually to directly test which subunit binds DNA. However, an AlphaFold3 structure prediction of the GAPS4^MAVP-R^ complex together with a random dsDNA molecule suggested that the dsDNA molecule is positioned within the GAPS4b-formed ring (Fig. 5b and Supplementary File S4). Therefore, we hypothesize that the predicted ring formed by two GAPS4b subunits can recognize an as-yet-unknown aberrant DNA, and that the GAPS4a phosphodiesterase subunits may then be positioned to access it.

Collectively, our results establish GAPS4 as a widespread system triggered by DNA damage, rendering it effective against diverse phages while sensitizing the bacteria to DNA-damaging antibacterial agents.

## Discussion

Numerous anti-phage defense systems have been described in recent years [5], yet in most cases, their trigger and activity remain unknown [2]. Here, we describe GAPS4, a widespread anti-phage defense system. We show that GAPS4 contains a domain belonging to the PD-(D/E)xK phosphodiesterase superfamily [38], which is required for its activity. Furthermore, we demonstrate that GAPS4 is triggered by attacking phages, antibiotic compounds, and antibacterial toxins that induce DNA damage, leading to host DNA degradation and cell death.

Anti-phage defense systems may respond to DNA damage by directly sensing aberrant DNA [41, 49, 50] and/or by indirect activation via the SOS response cascade, which is turned on by DNA damage [42, 43]. Although we did not identify the specific form of aberrant DNA that triggers GAPS4 protective activity in this work, our results imply that GAPS4 activation does not require the SOS response, suggesting that it can directly sense DNA damage. This notion is further supported by GAPS4’s ability to bind DNA *in vitro*. Notably, we found that GAPS4 protects against lytic and lysogenic phage attacks. Since lysogenic infections are not expected to incur DNA damage, we hypothesize that GAPS4 is triggered during such infections by sensing incoming linear phage DNA or changes in the chromosome during phage genome integration. Based on structural predictions of the GAPS4 complex, we hypothesize that a ring formed by two GAPS4b subunits can sense aberrant DNA, thereby positioning the phosphodiesterase-domain-containing GAPS4a subunits near it, and perhaps even activating it upon a conformational change. If correct, this model would indicate that the GAPS4 subunits function not as a toxin-antitoxin module but as a sensor-effector module, as previously demonstrated for other anti-phage defense systems [51]. The absence of toxicity when each GAPS4 subunit is expressed alone further supports the notion that GAPS4 is not a toxin–antitoxin system.

Although we demonstrated direct binding of the GAPS4 complex to DNA *in vitro*, we did not observe DNA cleavage using diverse forms of isolated and amplified DNA (not shown). We suspect that we either have yet to identify and provide the specific DNA species that activates GAPS4 *in vitro*, or that we are missing a cofactor that is present *in vivo*. Alternatively, purification of the complex may have rendered it inactive. Therefore, additional biochemical and structural work is required to determine the specific form of aberrant DNA sensed by GAPS4 and the activity of its phosphodiesterase domain.

The main takeaway from this work is that GAPS4 and possibly other anti-phage defense systems that sense DNA damage are potential double-edged swords. As we have demonstrated, GAPS4 sensitizes its bacterial host to antagonistic attacks that induce DNA damage, suggesting a trade-off between phage defense and susceptibility to interbacterial antagonism. Other anti-phage defense systems, such as CBASS, were recently shown to increase antibiotic sensitivity [52]. Another interesting example of a trade-off between phage defense and susceptibility to interbacterial antagonism was reported by Tsai *et al*., who demonstrated that bacterial lipopolysaccharides, which protect bacteria against interbacterial antagonism, can sensitize them to phages [53]. Although mediated by diverse mechanisms of action, the phenomenon of anti-phage defenses incurring a fitness trade-off when bacteria face non-phage antagonists may be common.

The proposed activation mechanism for GAPS4 is consistent with the recent observation that GAPS4 incurs an expression-controlled trade-off between the benefit of protection range and the cost of autoimmunity [54]. When overexpressed, it is more likely to detect random DNA damage in the bacterial genome, thereby degrading genomic DNA and leading to cell death. Importantly, the protective and sensitizing effects elicited by GAPS4 in this work were mainly demonstrated using GAPS4^RiKo^, which is encoded on a low-copy plasmid and controlled by its natural promoter, thereby avoiding the autoimmunity cost of overexpression. We further propose that GAPS4 may maintain relatively low expression not only because of its potential for autoimmunity but also because it is a double-edged sword that sensitizes the bacterium to antagonistic DNA-damaging agents and toxins.

Furthermore, sensing DNA damage to activate the nuclease activity of a PD-(D/E)xK effector domain appears to be a common theme in anti-phage defense systems [8, 55]. For example, the defense systems Shedu [49, 50] and Hachiman [41] were recently shown to target DNA upon sensing dsDNA ends or aberrant DNA states, nicking or cleaving dsDNA, respectively. Notably, upon overexpression, Hachiman was found to sensitize its host to the DNA-damaging agent nalidixic acid [41], as observed with GAPS4.

Interestingly, both GAPS4^MAVP-R^ and GAPS4^RiKo^ reside within MGEs. The former was identified within the cargo of a GMT island [13], whereas the latter is encoded within the Z-region of a P2-like prophage, which is a known hotspot containing diverse accessory gene cassettes in P2-like coliphages [56]. This location of GAPS4 homologs within MGEs could indicate a role for this system in MGE warfare rather than strictly in bacterial host immunity [57].

## Supplementary Material

gkag1104_Supplemental_Files

## Data Availability

All data related to the manuscript are available in the main text and the Supplementary Material files.

## Notes

Present address: School of Biological Sciences, National Institute of Science Education & Research, Bhubaneswar 752050, India

Present address: Training School Complex, Homi Bhabha National Institute, Mumbai 400094, India
