# Supplementary material for: The anti-phage defense system GAPS4 is a double-edged sword that sensitizes bacteria to DNA-damaging agents
Source: Nucleic Acids Res. 2026 Nov 10;54(21):gkag1104. doi: 10.1093/nar/gkag1104 (PMC13748975; doi:10.1093/nar/gkag1104)
Supplement: Supplementary file 1 [file gkag1104_supplemental_files.zip › Supplemental information_NAR_revision_1.pdf]

Supplemental information

The anti-phage defense system GAPS4 is a double-edged sword that sensitizes bacteria to DNA-damaging agents

Tridib Mahata, Katarzyna Kanarek, Anushree Haldar, Rameshkumar Marimuthu Ragavan, Guy Shur, Ruba Yehia, David Burstein, Yoni Haitin, Udi Qimron, and Dor Salomon

Figures S1-S6

Tables S1-S4

Dataset S1 caption

Files S1-S4 captions

References

Supplementary Figures

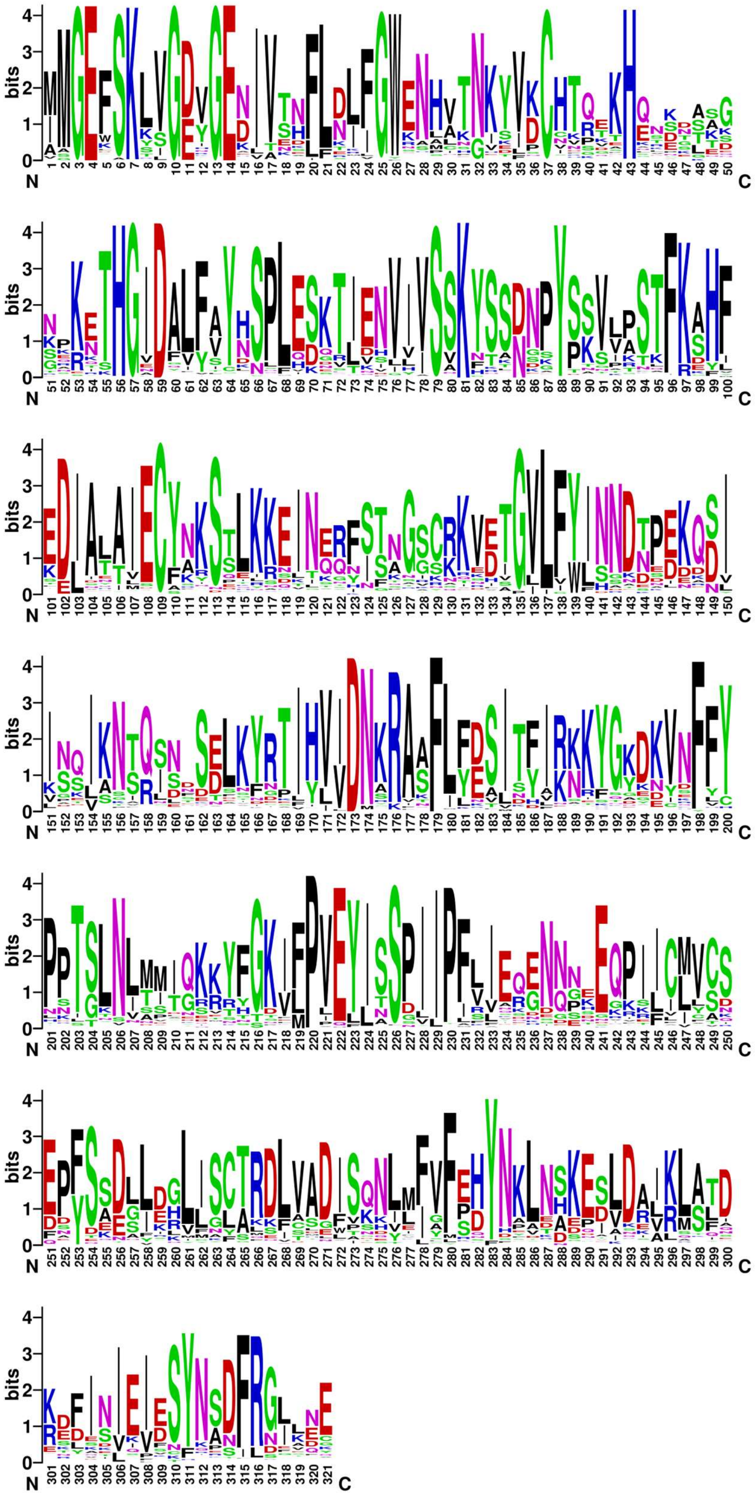

Fig. S1. Amino acid conservation of GAPS4a. Conservation logo based on a multiple sequence alignment of GAPS4a homologs. The position numbers correspond to the amino acids in WP_055466293.1.

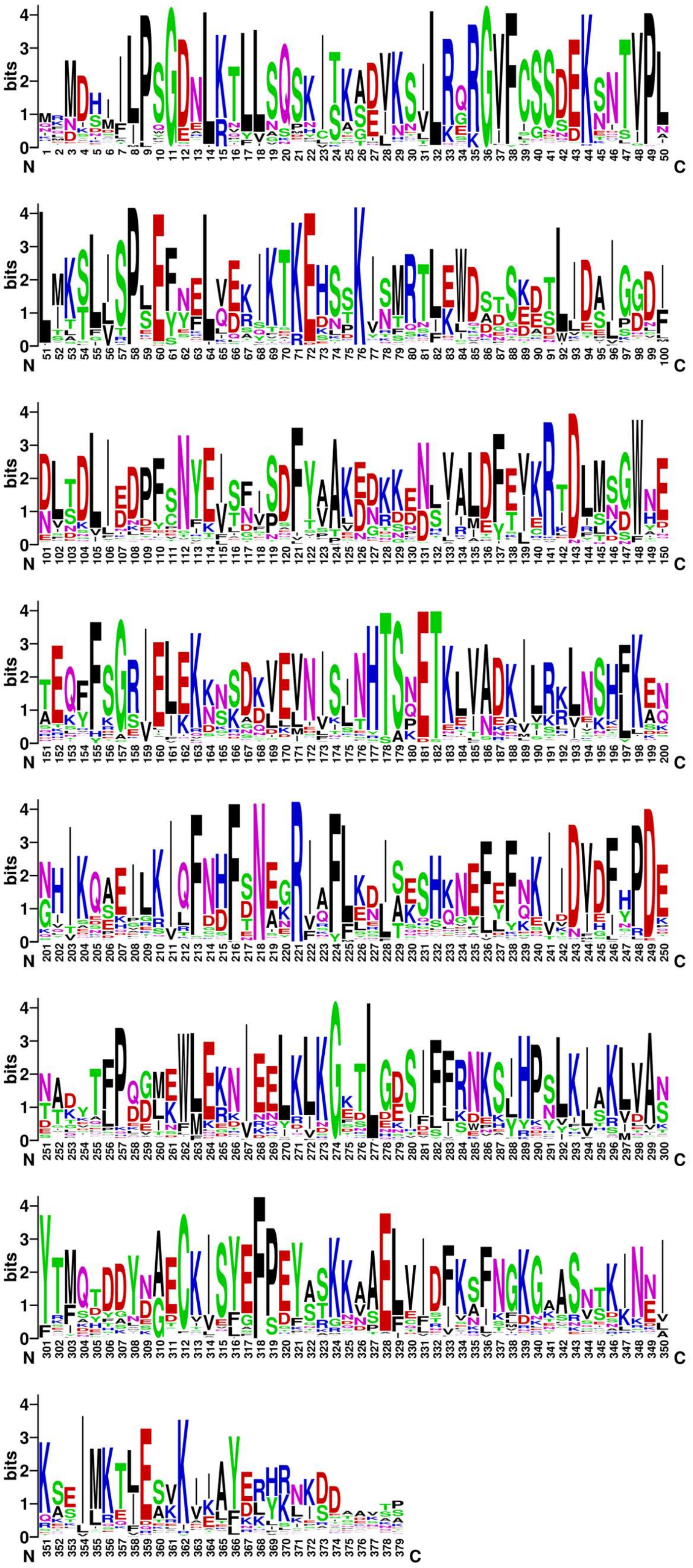

Fig. S2. Amino acid conservation of GAPS4b. Conservation logo based on a multiple sequence alignment of GAPS4b homologs. The position numbers correspond to the amino acids in WP_055466294.1.

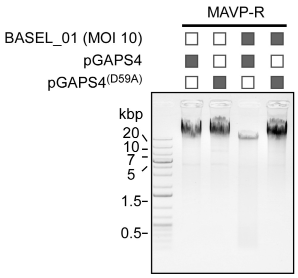

Fig. S3. GAPS4 degrades DNA upon BASEL_01 phage infection. Genomic DNA stability after the addition of BASEL_01 phage to the media of *E. coli* cultures expressing the indicated $GAPS4^{MAVP\text{-}R}$ variant from an arabinose-inducible plasmid. MOI, multiplicity of infection. The experiment was repeated three times with similar results. Results from a representative experiment are shown.

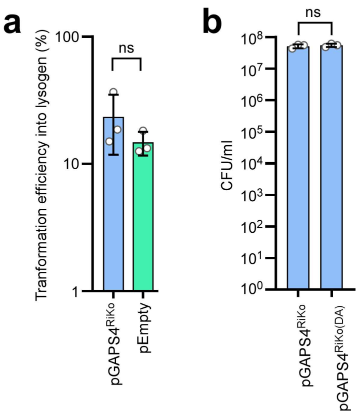

Fig. S4. **λ phage lysogens and T7 phage-mediated transduction do not induce GAPS4-mediated cell death. (a)** Transformation efficiency (percentage) of an empty plasmid (pEmpty) and a plasmid expressing $GAPS4^{RiKo}$ ($pGAPS4^{RiKo}$) into a λ lysogen *E. coli* strain. **(b)** Colony-forming units (CFU) of *E. coli* BW25113 Δ*trxA* containing a plasmid expressing the indicated $GAPS4^{RiKo}$ form, transduced with a T7 phage-based particle containing a plasmid conferring kanamycin resistance. For a and b, statistical significance between samples was calculated using an unpaired, two-tailed Student *t*-test; ns, no significant difference ($P > 0.05$). Data are shown as mean ± SD of 3 independent experiments.

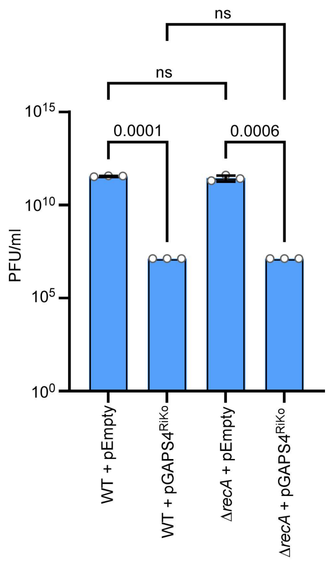

Fig. S5. SOS response is not required for GAPS4-mediated protection. Plaque-forming units (Pfus) of λ-vir phage upon infection of *E. coli* strains containing an empty plasmid (pEmpty) or a plasmid for the expression of $GAPS4^{RiKo}$ from its native promoter. Statistical significance between samples was calculated using one-way ANOVA with Sidak's multiple comparisons correction; ns, no significant difference ($P > 0.05$). Data are shown as mean ± SD of 3 independent experiments.

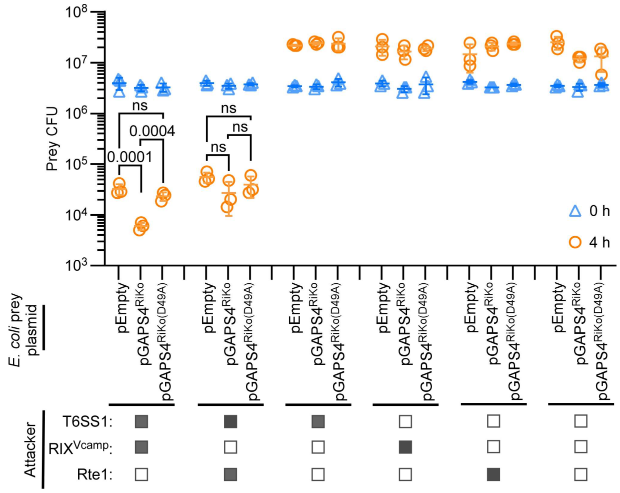

Fig. S6. GAPS4 sensitizes bacteria to specific T6SS effectors. Viability counts (colony forming units; CFU) of *E. coli* prey strains containing the indicated plasmids before (0 h) and after (4 h) co-incubation with *Vibrio parahaemolyticus* attacker strains lacking their endogenous T6SS1 antibacterial effectors, with either a functional (full square) or inactive (empty square) T6SS1, containing either an empty plasmid (empty square) or a plasmid for expression of the effectors RIX^{Vcamp} (WP_005534959.1) together with its cognate immunity gene, or Rte1 (WP_006958655.1) together with its cognate immunity gene and secreted adaptor (full square). Statistical significance between samples at the 4 h time point was assessed using one-way ANOVA with Tukey's multiple-comparison correction on log-transformed data. Data are shown as mean ± SD of 3 independent samples. ns, no significant difference ($P > 0.05$). The experiments were repeated three times with similar results. Results from a representative experiment are shown.

Supplementary Tables

Table S1. Bacterial strains used in this study.

Strain	Genotype	Source
Escherichia coli Neb5α	F⁻ f80*lacZ*DM15D*(lacZYA-argF)* U169 *deoR recA1 endA1 hsdR17* (r_K^-, m_K^+) *gal⁻ phoA supE44* l⁻ *thi⁻1 gyrA96 relA1*	NEB
Escherichia coli BW25113	*lacI*^{q}, rrnBT14, D*lacZ*wJ16, *hsd*R514, D*ara*BAD AH33, D*rha*BAD LD78	Lab collection
Escherichia coli BW25113 Δ*trxA*::kan	*lacI*^{q}, rrnBT14, D*lacZ*wJ16, *hsd*R514, D*ara*BAD AH33, D*rha*BAD LD79, *trxA* replaced with kanamycin resistance	Lab collection
Escherichia coli BW25113 Δ*trxA*	*lacI*^{q}, rrnBT14, D*lacZ*wJ16, *hsd*R514, D*ara*BAD AH33, D*rha*BAD LD79, *ΔtrxA*	Lab collection
Escherichia coli BW25113 Δ*recA::Kan*	*lacI*^{q}, rrnBT14, D*lacZ*wJ16, *hsd*R514, D*ara*BAD AH33, D*rha*BAD LD79, *trxA* replaced with kanamycin resistance	Lab collection
Escherichia coli BW25113 Δ*recA*	*lacI*^{q}, rrnBT14, D*lacZ*wJ16, *hsd*R514, D*ara*BAD AH33, D*rha*BAD LD79, *ΔrecA*	This study
Escherichia coli BW25113^{tetR}	*lacI*^{q}, rrnBT14, D*lacZ*wJ16, *hsd*R514, D*ara*BAD AH33, D*rha*BAD LD78, araB::T7RNAP-tetA	(1)
Escherichia coli TMB101	*Escherichia coli* BW25113, araB::T7RNAP-tetA, λ-cI857	This study
Escherichia coli DH5α λ-pir	F– φ80lacZΔ*M15* Δ(*lacZYA-argF*)*U169 recA1 endA1 hsdR17*(*rK– mK+*) *phoA supE44 λ– thi-1 gyrA96 relA1* Δ(*argF-lac*)169 pir+	Lab collection
Escherichia coli MG1655	F- lambda- ilvG- rfb-50 rph-1	Lab collection
Escherichia coli K12 MG1655 ΔRM	*Escherichia coli* K-12 MG1655 Δ*mrr-hsdRMS-mcrBC* Δ*mcrA* = ΔRM with pBR322_ΔPtet F(pifA::zeoR)	Alexander Harms (ETH Zurich)
Escherichia coli BL21 (DE3)	*fhuA2 [lon] ompT gal (λ DE3) [dcm]* Δ*hsdS λ DE3 = λ sBamHIo* Δ*EcoRI-B int::(lacI::PlacUV5::T7 gene1) i21* Δ*nin5*	Lab collection
Escherichia coli RiKo 2299/09	Wild-type isolate	Ulrich Dobrindt (University of Münster)
Vibrio parahaemolyticus RIMD 2210633	Wild-type isolate	Kim Orth (University of Texas Southwestern Medical Center)
Vibrio parahaemolyticus T6SS1⁺ effectorless	RIMD 2210633 Δ*hns/*Δ*tdhAS/* Δ*vp_rs06130/*Δ*vp_rs06745/vp_rs06875^{AAA}*	(2)

Vibrio parahaemolyticus T6SS1⁻ effectorless	RIMD 2210633 Δ*hns*/Δ*tdhAS*/ Δ*vp_rs06130*/Δ*vp_rs06745*/ *vp_rs06875*^{AAA}/Δ*hcp1*	(2)

Table S2. Plasmids used in this study.

Plasmid	Origin of replication	Antibiotic resistance	Comments	Source
pBAD33.1	ori15a	Chloramphenicol	L-arabinose-inducible expression vector	Addgene #36267
pGAPS4$^{MAVP\text{-}R}$	ori15a	Chloramphenicol	GAPS4 from *Vibrio parahaemolyticus* MAVP-R cloned in the pBAD33.1 MCS	(3)
pGAPS4$^{MAVP\text{-}R(D59A)}$	ori15a	Chloramphenicol	A D59A mutation introduced into pGAPS4$^{MAVP\text{-}R}$	This study
pGAPS4a$^{MAVP\text{-}R}$	ori15a	Chloramphenicol	GAPS4a from *Vibrio parahaemolyticus* MAVP-R in the pBAD33.1 MCS	This study
pGAPS4b$^{MAVP\text{-}R}$	ori15a	Chloramphenicol	GAPS4b from *Vibrio parahaemolyticus* MAVP-R in the pBAD33.1 MCS	This study
pRIX^{Vcamp}	ori15a	Chloramphenicol	*Vibrio campbellii* ATCC 25920 A8140_RS24680-A8140_RS24675 cloned under the arabinose promoter, in-frame with the C-terminal FLAG-tag	(4)
pRte1	ori15a	Chloramphenicol	*Vibrio coralliilyticus* ATCC BAA-450 operon comprised of VIC_RS26010-VIC_RS06695-VIC_RS26015 cloned under the arabinose promoter, in-frame with the C-terminal FLAG-tag	(4)
pC-0_v5	pSC101	Ampicillin	A low-copy-number plasmid	Addgene #124425
pGAPS4^{RiKo}	pSC101	Ampicillin	GAPS4 from *E. coli* RiKo 2299/09 cloned into pC-0_v5 along with 182 bp upstream containing the native promoter, and 235 bp downstream containing the native terminator sequences	This study
pGAPS4$^{RiKo(D49A)}$	pSC101	Ampicillin	A D49A mutation introduced into pGAPS4$^{RiKo(D49A)}$	This study

pMBP-cas1	pBR322	Kanamycin	Cas1 cloned in T7-His-TEV-MBP plasmid	(5)
pMBP-GAPS4^{MAVP-R}	pBR322	Kanamycin	GAPS4^{MAVP-R} cloned instead of Cas1 in the pMBP-cas1 plasmid, where GAPS4a is in-frame with the N-terminal MBP	This study
pBAD^{K}/Myc-His	pBR322	Kanamycin	An arabinose-inducible expression vector	(6)
pBAD^{K}/Myc-His-PoNe/i$^{Vp\ 12-\ 297/B}$	pBR322	Kanamycin	*Vibrio parahaemolyticus* 12-297/B VgrG1b with the downstream effector and immunity module PoNe/i, consisting of b5c30_rs14470-60 and the 3' gene, cloned in-frame with the C-terminal Myc-6xHis tag of the pBAD^{K}/Myc-His plasmid	(7)
pIYPE69	ori15a	Kanamycin	A plasmid containing a T7-packaging signal	(8)
PCP20	pSC101	Ampicillin	Encoding flippase	Lab collections
pCmLux2	pBBR1MCS4	Chloramphenicol	Broad-host-range bioluminescence reporter plasmid constitutively expressing the *luxCDABE* operon	(9)
pL(lexO)-GFP (pSOS)	pBR322-derived	Ampicillin	GFP reporter under the control of the *E. coli recA* SOS-responsive promoter (P*recA*); used to monitor SOS pathway activation	(10)

Table S3. DNA primers used in this study.

Primer name	Sequence (5'-> 3')'	Use
TM573F	TTAAGAAGGAGATATACATATGTCAG GAGAGAAATCG	Amplify pGAPS4$^{\text{MAVP-R}}$ for site-directed mutagenesis to construct pGAPS4$^{\text{MAVP-R(D59A)}}$
TM555R	CCTGAAATGTATGCATATTGTGCAGC AATACCATGCTGTTCTCGTTTTCC	
TM555F	GGAAAACGAGAACAGCATGGTATTG CTGCACAATATGCATACATTTCAGG	Amplify pGAPS4$^{\text{MAVP-R}}$ for site-directed mutagenesis to construct pGAPS4$^{\text{MAVP-R(D59A)}}$
TM572R	CATCCGCCAAAACAGCCAAGCTT	
TM559R	ATCCGCCAAAACAGCCAAGCTTTTAT TCATCGTTTAAGTTCCTAAAATTTGG	Amplify pGAPS4$^{\text{MAVP-R}}$ along with TM573F to construct pGAPS4a$^{\text{MAVP-R}}$
TM560F	TTAAGAAGGAGATATACATATGAATA ACGTTAGCACTTTCGTCC	Amplify pGAPS4$^{\text{MAVP-R}}$ along with TM572R to construct pGAPS4b$^{\text{MAVP-R}}$
IY508R	TGATGTAGCCGTCAAGTTGTCATAA	Amplify the pC-o_V5 backbone to construct pGAPS4^{RiKo}
IY139F	TGCTGCCACCGCTGAGCAAT	
TM712F	TGACAACTTGACGGCTACATCAGTTG TATTAGCTTACATATCTATGGCACAG	Amplify GAPS4^{RiKo} from *E. coli* strain RiKo 2299/09 to construct pGAPS4^{RiKo}
TM712R	TTATTGCTCAGCGGTGGCAGCATGT GCAGAGCATGAGACGCTATTTG	
AH33R	GAGTTGTAGACATACAAAGCAGCTAT TCCATGTGTCTTTTTAGC	Amplify pGAPS4^{RiKo} along with TM712F for site-directed mutagenesis to construct pGAPS4$^{\text{RiKo(D49A)}}$
AH33F	GCTAAAAAGACACATGGAATAGCTGC TTTGTATGTCTACAACTC	Amplify pGAPS4^{RiKo} along with TM712R for site-directed mutagenesis to construct pGAPS4$^{\text{RiKo(D49A)}}$
TM224F	GATAACGGATCCGAATTCGAGCG	Amplify pMBP-*cas1* backbone to construct pMBP-GAPS4$^{\text{MAVP-R}}$
TM224R	GGATTGGAAGTACAGGTTTTCCTCG	
TM604F	GGAAAACCTGTACTTCCAATCCATGT CAGGAGAGAAATCGAAGTCC	Amplify GAPS4$^{\text{MAVP-R}}$ from pGAPS4$^{\text{MAVP-R}}$ to construct pMBP-GAPS4$^{\text{MAVP-R}}$
TM604R	GCTCGAATTCGGATCCGTTATCTTAG GACGAGCCAGGAGGAG	
TM788F	ACCAAAAGGTTATTATATAGACGCCA TTGACGGCAATCCACTCAAC	
T4082	CATGGGCCAACTTTTATGGG	Amplify a 2,054-bp PCR product from *E. coli*, used in the gel shift assay
T4083	GCTGTTTACGGCGAGGAAAC	

Table S4. List of phages used in this study.

Phage	Family	Source
T4	*Myoviridae*	Lab collection
T5	*Siphoviridae*	Lab collection
T7	*Podoviridae*	Lab collection
λ-vir	*Siphoviridae*	Lab collection
P1-vir	*Myoviridae*	Lab collection
Basel 01	*Drexlerviridae; Braunvirinae*	(11)
Basel 02	*Drexlerviridae; Braunvirinae*	
Basel 03	*Drexlerviridae; Braunvirinae*	
Basel 04	*Drexlerviridae; Tempevirinae*	
Basel 05	*Drexlerviridae; Tempevirinae*	
Basel 06	*Drexlerviridae; Tempevirinae*	
Basel 07	*Drexlerviridae; Tempevirinae*	
Basel 08	*Drexlerviridae; Tempevirinae*	
Basel 09	*Drexlerviridae; Tempevirinae*	
Basel 10	*Drexlerviridae; Tempevirinae*	
Basel 11	*Drexlerviridae; Tempevirinae*	
Basel 12	*Drexlerviridae; Tunavirinae*	
Basel 13	*Drexlerviridae; Tunavirinae*	
Basel 14	*Siphoviridae*	
Basel 15	*Siphoviridae*	
Basel 16	*Siphoviridae*	
Basel 17	*Siphoviridae*	
Basel 18	*Siphoviridae*	
Basel 20	*Siphoviridae, Queuovirinae*	
Basel 21	*Siphoviridae, Queuovirinae*	
Basel 22	*Siphoviridae, Queuovirinae*	
Basel 23	*Siphoviridae, Queuovirinae*	
Basel 24	*Siphoviridae, Queuovirinae*	
Basel 25	*Siphoviridae, Queuovirinae*	
Basel 26	*Demerecviridae; Markadamsvirinae*	
Basel 27	*Demerecviridae; Markadamsvirinae*	
Basel 28	*Demerecviridae; Markadamsvirinae*	
Basel 29	*Demerecviridae; Markadamsvirinae*	
Basel 30	*Demerecviridae; Markadamsvirinae*	
Basel 31	*Demerecviridae; Markadamsvirinae*	
Basel 32	*Demerecviridae; Markadamsvirinae*	

Basel 33	*Demerecviridae; Markadamsvirinae*	
Basel 34	*Demerecviridae; Markadamsvirinae*	
Basel 35	*Myoviridae; Tevenvirinae*	
Basel 36	*Myoviridae; Tevenvirinae*	
Basel 37	*Myoviridae; Tevenvirinae*	
Basel 38	*Myoviridae; Tevenvirinae*	
Basel 39	*Myoviridae; Tevenvirinae*	
Basel 40	*Myoviridae; Tevenvirinae*	
Basel 41	*Myoviridae; Tevenvirinae*	
Basel 42	*Myoviridae; Tevenvirinae*	
Basel 43	*Myoviridae; Tevenvirinae*	
Basel 44	*Myoviridae; Tevenvirinae*	
Basel 45	*Myoviridae; Tevenvirinae*	
Basel 46	*Myoviridae; Tevenvirinae*	
Basel 47	*Myoviridae; Tevenvirinae*	
Basel 48	*Myoviridae; Vequintavirinae*	
Basel 49	*Myoviridae; Vequintavirinae*	
Basel 50	*Myoviridae; Vequintavirinae*	
Basel 51	*Myoviridae; Vequintavirinae*	
Basel 52	*Myoviridae; Vequintavirinae*	
Basel 53	*Myoviridae; Vequintavirinae*	
Basel 54	*Myoviridae; Vequintavirinae*	
Basel 55	*Myoviridae; Vequintavirinae*	
Basel 56	*Myoviridae; Vequintavirinae*	
Basel 57	*Myoviridae; Vequintavirinae*	
Basel 58	*Myoviridae; Vequintavirinae*	
Basel 59	*Myoviridae; Vequintavirinae*	
Basel 60	*Myoviridae; unclassified*	
Basel 61	*Myoviridae; unclassified*	
Basel 62	*Myoviridae; unclassified*	
Basel 63	*Myoviridae; Ounavirinae*	
Basel 64	*Autographiviridae; Studiervirinae*	
Basel 65	*Autographiviridae; Studiervirinae*	
Basel 66	*Autographiviridae; Studiervirinae*	
Basel 67	*Autographiviridae; Studiervirinae*	
Basel 68	*Autographiviridae; Studiervirinae*	
Basel 69	*Schitoviridae; Enquatrovirinae*	
λ-CI857	*Siphoviridae*	(12)

Supplementary Datasets

Dataset S1. A list of GAPS4 homologs identified in this study

Supplementary Files

File S1. The concatenated amino acid sequences of GAPS4a and GAPS4b homologs identified in this study

File S2. AlphaFold3 structure prediction for GAPS4$^{MAVP\text{-}R}$

File S3. AlphaFold3 structure prediction for GAPS4^{RiKo}

File S4. AlphaFold3 structure prediction for GAPS4$^{MAVP\text{-}R}$ with a random dsDNA molecule

Supplementary References

1. Yosef,I., Goren,M.G. and Qimron,U. (2012) Proteins and DNA elements essential for the CRISPR adaptation process in Escherichia coli. *Nucleic Acids Res.*, **40**, 5569–5576.

2. Jana,B., Keppel,K., Fridman,C.M., Bosis,E. and Salomon,D. (2022) Multiple T6SSs, mobile auxiliary modules, and effectors revealed in a systematic analysis of the Vibrio parahaemolyticus pan-genome. *mSystems*, **7**, e00723-22.

3. Mahata,T., Kanarek,K., Goren,M.G., Marimuthu Ragavan,R., Bosis,E., Qimron,U. and Salomon,D. (2024) Gamma-Mobile-Trio systems are mobile elements rich in bacterial defensive and offensive tools. *Nat. Microbiol.*, **9**, 3268–3283.

4. Kanarek,K., Fridman,C.M., Bosis,E. and Salomon,D. (2023) The RIX domain defines a class of polymorphic T6SS effectors and secreted adaptors. *Nat. Commun.*, **14**, 4983.

5. Yosef,I., Mahata,T., Goren,M.G., Degany,O.J., Ben-Shem,A. and Qimron,U. (2023) Highly active CRISPR-adaptation proteins revealed by a robust enrichment technology. *Nucleic Acids Res.*, **51**, 7552–7562.

6. Salomon,D., Gonzalez,H., Updegraff,B.L. and Orth,K. (2013) Vibrio parahaemolyticus Type VI secretion system 1 Is activated in marine conditions to target bacteria, and is differentially regulated from system 2. *PLoS One*, **8**, e61086.

7. Jana,B., Fridman,C.M., Bosis,E. and Salomon,D. (2019) A modular effector with a DNase domain and a marker for T6SS substrates. *Nat. Commun.*, **10**, 3595.

8. Yosef,I., Goren,M.G., Globus,R., Molshanski-Mor,S. and Qimron,U. (2017) Extending the Host Range of Bacteriophage Particles for DNA Transduction. *Mol. Cell*, **66**, 721–728.

9. Fridman,C.M., Cohen,H., Keppel,K., Tejada-Arranz,A., Basler,M., Gerlic,M. and Salomon,D. (2026) *Aeromonas* adhesins facilitate kin and non-kin attachment to enable T6SS-mediated antagonism in liquid. *bioRxiv*, 10.64898/2026.01.27.701733.

10. Dwyer,D.J., Kohanski,M.A., Hayete,B. and Collins,J.J. (2007) Gyrase inhibitors induce an oxidative damage cellular death pathway in Escherichia coli. *Mol. Syst. Biol.*, **3**, MSB4100135.

11. Maffei,E., Shaidullina,A., Burkolter,M., Heyer,Y., Estermann,F., Druelle,V., Sauer,P., Willi,L., Michaelis,S., Hilbi,H., *et al.* (2021) Systematic exploration of Escherichia coli phage-host interactions with the BASEL phage collection. *PLoS Biol.*, **19**, e3001424.

12. Edgar,R. and Qimron,U. (2010) The Escherichia coli CRISPR system protects from λ lysogenization, lysogens, and prophage induction. *J. Bacteriol.*, **192**, 6291–6294.
